# Influence of Perch-Provision Timing on Anxiety and Fearfulness in Laying Hens

**DOI:** 10.3390/ani13193003

**Published:** 2023-09-23

**Authors:** Mallory G. Anderson, Alexa M. Johnson, Leonie Jacobs, Ahmed B. A. Ali

**Affiliations:** 1 Department of Animal and Veterinary Sciences, Clemson University, Clemson, SC 29634, USA; mga5@clemson.edu (M.G.A.); ajohn43@clemson.edu (A.M.J.); 2 Department of Animal and Poultry Sciences, Virginia Tech, Blacksburg, VA 24061, USA; jacobsl@vt.edu; 3 Animal Behavior and Management, Department of Veterinary Hygiene and Management, Faculty of Veterinary Medicine, Cairo University, Giza 12211, Egypt

**Keywords:** laying hen, behavior, attention bias, tonic immobility, perch

## Abstract

**Simple Summary:**

Perch access and age during access to perches may impact laying hen welfare. Our study aimed to determine the effects of early or late access to perches on behavioral measures of anxiety (AB: attention bias test) and fearfulness (TI: tonic immobility test) in laying hens. Pullets were housed in pens with or without access to perches until 17 weeks of age, at which point perch access either continued or was removed until 37 weeks of age, resulting in four treatments: continuous perch access (CP: 0–37 weeks), early perch access (EP: 0–17 weeks), late perch access (LP: 17–37 weeks), no perch access (NP). AB was performed at 21 and 37 weeks of age, and TI was performed at 20, 25, and 37 weeks of age. CP hens showed reduced anxiety and fearfulness, benefiting animal welfare, while NP hens showed increased anxiety and fearfulness. LP hens required around 16 weeks to adapt to the addition of perches in their environment, indicated by increased anxiety and fearfulness at 20 weeks of age that dissipated by week 37 of age. Removing perches in the EP pens resulted in increased fear and anxiety, which also disappeared by week 37 of age. Perch access benefits animal welfare, and removing or preventing access should be avoided.

**Abstract:**

Perches can enhance laying hen welfare, but their effectiveness might be age-dependent. We investigated early and late perch access effects on anxiety and fear in pullets through attention bias (AB) and tonic immobility (TI) tests. Pullets (*n* = 728) were raised with or without multi-level perches: CP (continuous perch access: 0–37 weeks), EP (early perch access: 0–17 weeks), LP (late perch access: 17–37 weeks), and NP (no perch access). AB was conducted in weeks 21 and 37 (*n* = 84/week), and TI was performed in weeks 20, 25, and 37 (*n* = 112/week). CP hens fed quicker than EP, LP, and NP in AB at weeks 21 and 37 (*p* ≤ 0.05). CP and NP feeding latencies were stable, while EP and LP fed faster at week 37 (*p* ≤ 0.05). CP had the shortest TI at week 20 (*p* < 0.05). CP and LP had the shortest TI in weeks 25 and 37 (all *p* ≤ 0.05). Unlike NP, CP reduced anxiety and fear. Adding perches during laying (LP) raised anxiety at week 21, adapting by week 37, and removing pre-laying perches (EP) worsened fear at weeks 20 and 25 and anxiety at week 21, recovering by week 37. Adding or removing perches prior to the lay phase increased fear and anxiety, an effect that disappeared by week 37 of age. Our study indicates that continuous perch access benefits animal welfare compared to no perch access at all.

## 1. Introduction

The evaluation of affective states can be used to improve animal welfare. Negative affective states, such as chronic anxiety and fear, raise major welfare concerns because while adaptive to survival, these negative affective states can lead to excessive responses to routine husbandry practices and a decreased ability to cope with environmental change in production settings. For example, extreme fear can result in panicked behavioral responses in laying hens in response to an unusual stockperson behavior, which in turn can lead to piling and suffocation [1]. Furthermore, excessive fear can cause increased sensitivity to stress, poor feed intake, low body weight, and decreased production [2,3]. Although difficult to distinguish, excessive anxiety may have similar negative consequences, as anxiety itself has been defined as a persistent, excessive, and inappropriate emotional state that triggers physiological and behavioral responses lacking adaptive value [4]. Therefore, it is important for animal welfare and productivity to keep anxiety and fear low. 

One approach to limit negative affective states is by providing housing conditions that meet motivational needs. For example, laying hens are highly motivated to perch even early in life, and fulfilling this motivation likely improves their affective states, although not directly assessed [5,6]. Laying hens housed in conventional cages were more fearful and showed lower antibody levels early in life compared to hens in enriched, cage-free housing environments [7]. Inducing positive experiences during the pullet phase is important, as behaviors become more rigid later in life after the ontogenetic period has passed [8]. For instance, pullets reared in complex aviary systems that were subsequently transitioned to barren cage environments at a reproductive age were less fearful than those reared in barren cage environments throughout, indicating that environmental complexity during rearing can reduce fearfulness later in life [9]. In addition, providing environmental complexity (i.e., perches) during rearing can improve musculoskeletal health [10], which in turn could benefit affective states due to their better physical ability to perch [11,12]. 

Adverse early life experiences, such as the inability to perform highly motivated behaviors, could have long-term negative impacts on laying hen cognition and behavior. Rearing pullets without perches impaired the spatial cognitive skills of the adult hen in a spatial cognition test compared to those reared with perches [13]. Hens refine their spatial skills as young pullets through practice, so preventing this could lead to impaired spatial cognition and an inability to successfully navigate their environment as they are moved to the laying hen facility [13,14]. Pullets given access to perches from 0 to 8 weeks of age jumped to higher perches compared to those not given access to perches until 8 weeks of age [13]. Although effects on cognition and behavior are determined, it is unknown whether early access to perches impacts pullet and laying hen emotion and affect.

The loss or gain of perches when transitioning from the pullet to the layer phase may impact anxiety and fearfulness. The removal of perches after the pullet phase leads to changes in behavior related to frustration and boredom because behavioral needs are not met [15,16,17], which could increase levels of anxiety and fearfulness. Laying hens show frustration-related behaviors, such as increased restlessness and attempted take-offs when access to perches is prevented compared to hens allowed access to perches [18]. It is possible that the effects of losing perch access may be more detrimental to animal welfare than not having any perches at all, but this has not been previously tested.

Anxiety levels can be assessed through attention bias (AB) testing, with AB referring to the differential allocation of attentional resources towards one stimulus compared to others [19]. For example, animals in anxious states exhibit increased attentional bias towards a potential threat, where more time spent focusing on a perceived threat compared to neutral or positive stimuli indicates increased anxiousness [20]. While anxiety is an affect-mediated response to potentially dangerous situations influenced by previous life experiences, fear is a short-term response to an immediate threat [21]. The behavioral responses of fear and their intensity, either rational or irrational, result from gene–environment interactions during the animal’s development and provide insight into their ability to cope with presently dangerous stimuli [21]. To measure fearfulness in poultry, a tonic immobility (TI) test is often used, which uses the prey species’ freezing response [22]. Longer tonic immobility durations positively correlate with increased fearfulness [23,24,25,26]. Although anxiety and fear responses can look similar, the two emotions can be opposing and are not always aligned [3,7,27]. Therefore, AB and TI tests could provide valuable insights into laying hen anxiety and fear levels in response to housing environments, as well as giving insight to the distinct emotional states. 

The use of AB and TI tests to evaluate anxiety and fearfulness could provide a better understanding of the impacts of perch provision and its timing on laying hen affective state and welfare. Our objective was to investigate the effects of early and late access to perches on anxiety and fearfulness in laying hens. Pullets were housed either with or without multi-tier perches from 0–37 weeks of age, and half of them experienced a loss or gain of perches at 17 weeks of age. We hypothesized that birds housed without any perch access would have the highest levels of anxiety and fearfulness, followed by those reared with perches that were subsequently taken away during the laying phase, then birds reared without perches that were later added to the environment, with birds housed with perches throughout the entire trial having the lowest levels of anxiety and fearfulness. 

## 2. Materials and Methods

### 2.1. Ethics

This experiment was approved by Clemson University’s Institutional Animal Care and Use Committee (protocol #: AUP2021-0068).

### 2.2. Animal and Housing

This experiment was conducted in a ventilation- and temperature-controlled poultry house at the Morgan Poultry Center, Clemson, South Carolina, USA, from December 2021 to August 2022. Day-old Hy-Line^®^ brown chicks (*n* = 728) were randomly allocated across 28 pens (26 birds/pen). Each pen was 5.04 m^2^ with approximately 7.6 cm deep clean pine wood shavings covering the floor. For the first 3 weeks, the heat was provided by a focal electric brooder per pen and a gas-fired brooder for the entire house. The temperature was initially set at 35–36.1 °C at day 0, then progressively reduced by 3–4 °C every week until 3 weeks of age, when brooders were removed. The temperature was reduced weekly until 6 weeks of age to 21.1°F, then maintained until the end of the study, following the standard breed guidelines [28]. Feed and water were provided ad libitum. From 0 to 3 weeks, the feed was provided in tube feeders and water in gallon drinkers. For the first week of life, supplementary feed trays were provided. After 3 weeks, feed was provided in circular hanging feeders, and water was available in automatic cup drinkers. The light was provided by a single 60-watt incandescent overhead lightbulb per pen, and pens were kept on a decreasing lighting schedule starting at 20 L:4 D cycle at 1 week old and decreased by increments of either 1.5 or 2 h until 10 L:14 D from 7 weeks of age to the end of the study (Hy-Line, 2022). During week 6 of age, all birds were neck tagged (GST15, Ketchum Manufacturing INC. ON, Canada) for individual identification. 

### 2.3. Treatments

During the rearing phase (0–17 weeks of age), pullets were either housed in pens with multi-tier perches (*n* = 14 pens) or without perches (*n* = 14 pens). At 17 weeks of age, birds within a pen were moved to a new pen so that their access to perches during the lay phase (17–37 weeks of age) was either removed or remained the same. Thus, all birds were exposed to the same level of stress from placement into a new setting simulating the pullet transfer from the rearing to the laying facility in the industry. This resulted in four treatments: continuous perch access from 0–37 weeks of age (CP; *n* = 7 pens); early perch access only during the rearing phase from 0–17 weeks of age (EP; *n* = 7 pens); late perch access only during lay phase from 17–37 weeks of age (LP; *n* = 7 pens); and no perch access from 0–37 weeks of age (NP; *n* = 7 pens). The adjustable perches were built from 5 × 5cm pressure-treated wooden lumber. Each perch structure contained 3 rungs of varying height, each 165.1 cm in length, resulting in 495.3cm of total perching space and approximately 19 cm of perch space per bird. The rungs were 38.1 cm, 62.2 cm, and 88.4 cm high, with a 12.7 cm distance between each perch rung.

### 2.4. Attention Bias Test

The AB test followed a group testing approach described by Campbell et al. and Anderson et al. [7,29] on three randomly selected birds per pen at “onset of lay” weeks 21 (*n* = 84; hen-day% = 81.85 ± 4.68%) and “peak-lay” 37 (*n* = 84; hen-day% = 94.52 ± 1.12%) of age. All 3 birds per pen were tested simultaneously. Two observers performed the AB test in a room adjacent to the main poultry house in a testing arena constructed of wire fencing (140 L × 132 W × 94 H cm) with pine shavings on the floor and a feeder containing poultry feed. Once the three birds were placed in the arena, a conspecific alarm call signaling a ground predator was played for 8s. Immediately following the alarm call, latencies to begin feeding (s) and the occurrence of vigilance behaviors during the first 30 s were recorded. Four vigilance behaviors were recorded (freezing, neck stretching, looking around, and erect posture) as either observed (1) or not observed (0) within the first 30 s of testing and summed to obtain a vigilance score for each individual bird ranging from 0 (no vigilance behavior observed) to 4 (all vigilance behaviors observed at least once), as previously described by [7,29]. Latencies to begin and resume feeding were recorded following the methodologies described by [7]. Birds from the first round of AB testing were identified by neck tag number and not tested again during the second round of AB testing. Individual identification between the birds during the AB test was possible by marking the birds with livestock spray (Quik Shot Livestock Marker, LA-CO Industries Inc., IL, USA). Table 1 summarizes the AB testing method and is adapted from Campbell et al. [7]. For more details on the attention bias testing methods, see [7,29]. 

### 2.5. Tonic Immobility Test

Tonic immobility (TI) was performed by two observers in the center area of the poultry house. At weeks 20 (onset of lay; hen-day% = 80.23 ± 5.85%), 25 (early-lay; hen-day% = 90.89 ± 3.47%), and 37 of age (peak-lay; hen-day% = 94.52 ± 1.12%), four randomly selected birds per pen (*n* = 128) were tested for TI as described by [29,30]. The birds selected for the TI test were not the same as those selected for the AB test. Similar to AB testing, individual birds were TI tested only once during the trial. TI was induced by the handler placing the bird on its back into a V-shaped cradle, then placing one hand over the sternum and the other over the head. After 15 s, the handler removed their hands from the bird, stepped out of its line of sight, and recorded latency until the righting response (TI duration [s]). If the bird attempted to right itself within 10 s of the handler removing their hands, the handler attempted to induce TI again by repeating the technique, with a maximum of three induction attempts. If TI could not be induced, the bird received a minimum latency score of 0 s. If the bird remained in TI for the full testing period (5 min), the bird received a maximum latency score of 300 s. Inter-observer reliability was calculated during a 3-day training period when the two observers performed AB and TI alternatively on the same 40 birds that were not included in the current study. Inter-observer reliability was calculated using Cohen’s kappa agreement coefficient (κ), following [31], using the “cohen.kappa” function in the “psych” package, and intra-observer agreement was considered good when Kappa exceeded 0.90 [Kappa = 0.96 (*p* < 0.001); 95% CI (0.90, 0.99)]. 

### 2.6. Statistical Analysis

Data were analyzed using the R software (version 3.3.1) with the package “stats” (R Core Team, 2013). To test for the main effects of treatment (CP, EP, LP, and NP) and the age of the birds (TI: 20, 25, and 37 weeks; AB: 21 and 37 weeks) on each variable, generalized linear mixed-effects models (GLMMs) were conducted using the “lme4” package (Bates, et al., 2014). In each GLMM, the interaction term between main effects was also tested as fixed effects, and bird ID and pen as random effects, with the family set to “Quasibinomial” for proportion data and “Poisson” for the other data. Tukey’s HSD multiple comparison procedure was used for post-hoc comparisons using the “multcomp” package [32]. The “DHARMa” package was used for proportion data (i.e., percentage of birds feeding and resumed feeding) to test residual distribution and assumptions for GLMM, while the Shapiro–Wilk test was utilized (i.e., TI duration (s) and time to begin and resume feeding (s)) for the normality analysis of the model residuals. Statistical significance was set at *p* < 0.05. Descriptive statistics were calculated using the “psych package”, and data are presented as mean ± standard error of the mean (SEM).

## 3. Results

### 3.1. Attention Bias Test

#### 3.1.1. Latency to Begin Feeding 

**Figure 1:**
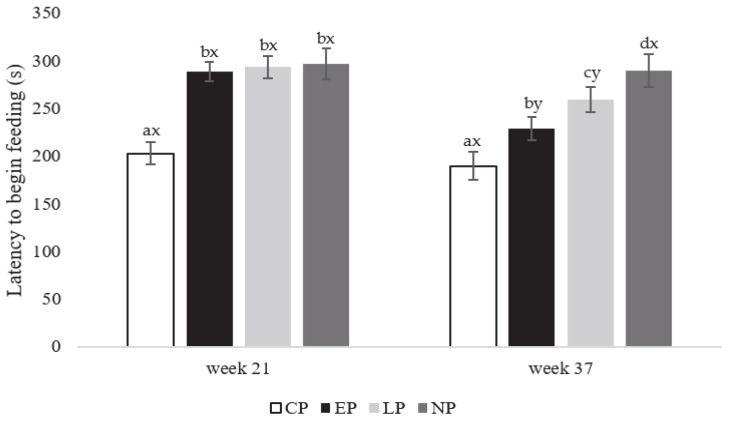
Latency to begin feeding (0–300 s) for laying hens in CP (continuous perch), EP (early perch), LP (later perch), and NP (no perch) housing environments during the attention bias test at onset of lay at week 21 and peak-lay at 37 of age (*n* = 112 hens/week). ^a–c^ Different superscripts indicate statistically significant differences between treatments within the same week at *p* < 0.05. ^x–z^ Different superscripts indicate statistically significant differences between weeks within the same treatment at *p* < 0.05.

At the onset of lay (week 21 of age), CP hens began feeding faster than EP, LP, and NP hens (F_3,80 =_ 235.23; *p* = 0.003; Figure 1). At peak-lay (week 37 of age; F_3,80_ = 544.19; *p* = 0.001), CP hens began feeding faster than EP hens (*p* = 0.021), while the latter fed faster than LP hens (*p* = 0.016), and LP hens faster than NP hens (*p* = 0.017; Figure 1). EP and LP hens (F_1,40_ = 196.85; *p* = 0.023) began feeding faster at week 37 compared to week 21 (*p* = 0.021 and 0.031, respectively; Figure 1), but no other post-hoc differences within treatment were observed. 

#### 3.1.2. Latency to Resume Feeding

**Figure 2:**
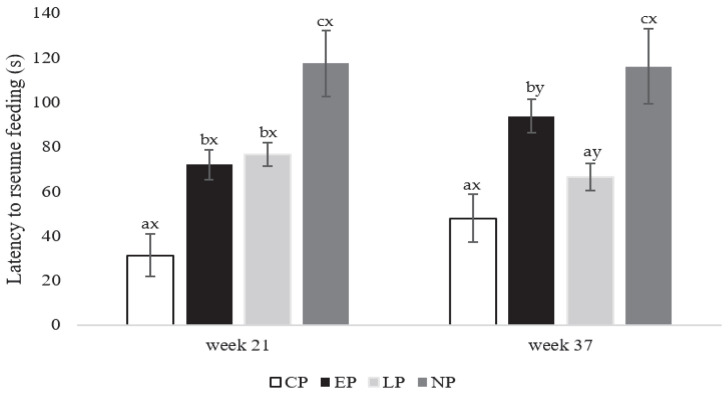
Latency to resume feeding (0–120 s) expressed as (mean ± SEM) for laying hens in CP (continuous perch), EP (early perch), LP (late perch), and NP (no perch) housing environments during the attention bias test at the onset of lay at week 21 and peak-lay at week 37 of age (*n* = 112 hens/week). ^a–c^ Different superscripts indicate statistically significant differences between treatments within the same week at *p* < 0.05. ^x–z^ Different superscripts indicate statistically significant differences between weeks within the same treatment at *p* < 0.05.

At week 21 of age (onset of lay), CP hens resumed feeding faster than EP and LP hens, with the longest latency to resume feeding observed in NP hens (F_3,80_ = 463.85; *p* = 0.001; Figure 2). Peak-lay at week 37 of age (F_3,80_ = 301.85; *p* = 0.002; Figure 2), CP and LP hens resumed feeding faster compared to EP hens (*p*= 0.021 and 0.032, respectively), with EP hens resuming feeding faster than NP hens (*p* = 0.029; Figure 2). Within treatment (F_1, 40_ = 124.46; *p* = 0.031; Figure 2), EP hens resumed feeding faster at week 21 compared to week 37 (*p* = 0.026), and LP hens resumed feeding faster at peak-lay compared to during the onset of lay (*p* = 0.019; Figure 2); however, no differences were observed between weeks 21 and 37 in latency to resume feeding for CP and NP hens.

#### 3.1.3. Percentage of Birds to Begin and Resume Feeding

**Figure 3:**
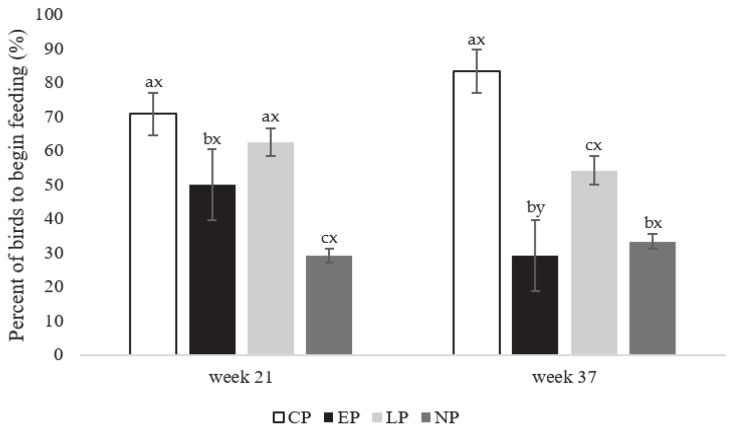
Percentage (%) of laying hens (expressed as mean ± SEM) observed to begin feeding from CP (continuous perch), EP (early perch), LP (late perch), and NP (no perch) housing environments during the attention bias test at onset of lay at week 21 and peak-lay at week 37 of age (*n* = 112 hens/week). ^a–c^ Different superscripts indicate statistically significant differences between treatments within the same week at *p* < 0.05. ^x–z^ Different superscripts indicate statistically significant differences between weeks within the same treatment at *p* < 0.05.

More birds from CP and LP pens began feeding compared to EP (F_3,80_ = 399.23; *p* = 0.021; Figure 3) and NP birds the onset of lay at week 21 (*p* = 0.016, and 0.023, respectively; Figure 3). While at peak-lay in week 37 (F_3,80_ = 423.26; *p* = 0.026; Figure 3), more CP birds began feeding than EP (*p* = 0.001), NP (*p* = 0.001), and LP birds (*p*= 0.026), more LP birds were observed to begin feeding than EP and NP (*p* = 0.033, and 0.036, respectively; Figure 3). EP pens had more birds feeding at week 21 compared to week 37 (F_1, 40_ = 99.56; *p* = 0.036; Figure 3), with no observed differences between weeks for other treatments. 

**Figure 4:**
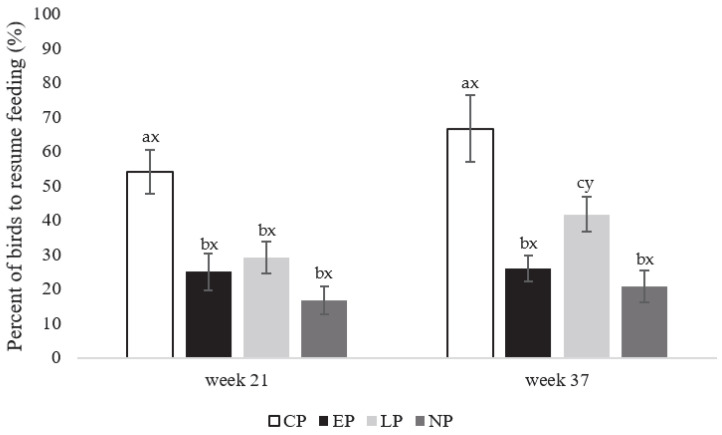
Percentage (%) of laying hens (expressed as mean ± SEM) observed to resume feeding from CP (continuous perch), EP (early perch), LP (late perch), and NP (no perch) housing environments during the attention bias test at onset of lay at week 21 and peak-lay at week 37 of age (*n* = 112 hens/week). The timer was reset to zero after the second alarm call was played to record latency to resume feeding. ^a–c^ Different superscripts indicate statistically significant differences between treatments within the same week at *p* < 0.05. ^x–z^ Different superscripts indicate statistically significant differences between weeks within the same treatment at *p* < 0.05.

More birds from CP pens resumed feeding (F_3,80_ = 248.52; *p* = 0.019) compared to birds from LP, EP, and NP pens in week 21 (*p* = 0.026, 0.021, and 0.017, respectively; Figure 4). Similarly, more CP birds resumed feeding (F_3,80_ = 301.26; *p* = 0.023) than EP, NP, and LP pens in week 37 (*p* = 0.013, 0.019, and 0.029, respectively; Figure 4), while the LP group showed more birds resuming feeding than EP (*p*= 0.032) and NP (*p* = 0.029) pens. Within treatment (F_1, 40_ = 108.32; *p* = 0.031), more birds from LP pens resumed feeding at week 37 compared to week 21 (*p* = 0.027), with no observed differences between weeks for other treatments (Figure 4). 

#### 3.1.4. Vigilance Behavior

**Figure 5:**
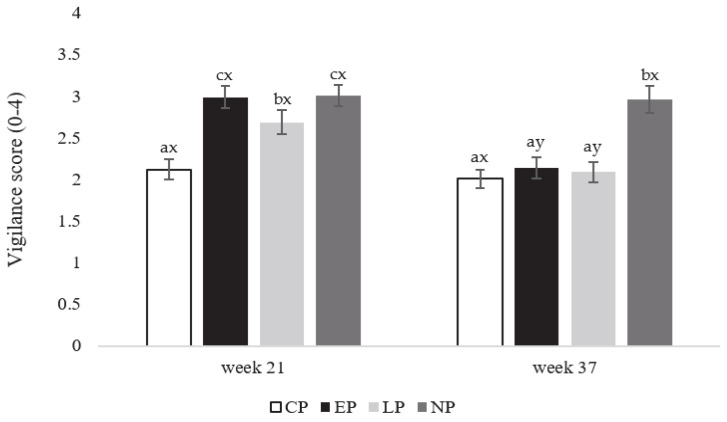
Vigilance behavior scores (expressed as means ± SEM) for laying hens in CP (continuous perch), EP (early perch), LP (late perch), and NP (no perch) housing environments during AB testing at onset of lay at week 21 and peak-lay at week 37 of age (*n* = 84 hens/week). ^a–c^ Different superscripts indicate statistically significant differences between treatments within the same week at *p* < 0.05. ^x–z^ Different superscripts indicate statistically significant differences between weeks within the same treatment at *p* < 0.05.

Vigilance behavior scores differed between treatments at the onset of lay in week 21 (F_3,80_ = 98.36; *p* = 0.033), with NP and EP hens having the highest scores compared to LP hens (*p* = 0.036 and 0.032, respectively), with the lowest vigilance score seen in CP hens (*p* = 0.023 and 0.021, respectively; Figure 5). At peak-lay in week 37 (F_3,80_ = 89.58; *p* = 0.026), NP hens had the highest vigilance score compared to the other treatment groups (*p* = 0.019 (CP), 0.022 (EP), 0.031 (LP); Figure 5). Between weeks (F_1, 40_ = 112.69; *p* = 0.029), EP and LP hens had the highest vigilance scores at week 21 compared to 37 (*p* = 0.021 and 0.036, respectively), with no differences in vigilance scores between weeks in the other treatment groups (Figure 5).

### 3.2. Tonic Immobility Test

#### 3.2.1. Tonic Immobility Duration

**Figure 6:**
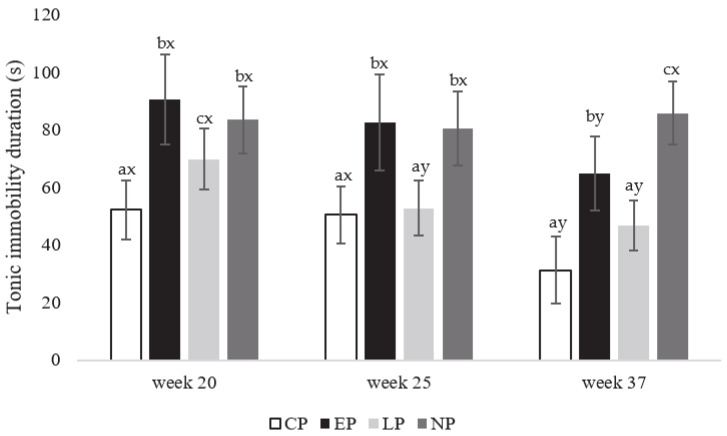
Tonic immobility duration (0–300 s) expressed as (mean ± SEM) for laying hens in CP (continuous perch), EP (early perch), LP (late perch), and NP (no perch) housing environments at the onset of lay at week 21, early-lay at week 25, and peak-lay at 37 weeks of age (n = 112 hens/week). ^a–c^ Different superscripts indicate statistically significant differences between treatments within the same week at *p* < 0.05. ^x–z^ Different superscripts indicate statistically significant differences between weeks within the same treatment at *p* < 0.05.

CP hens had the shortest TI duration compared to EP, LP, and NP hens at the onset of lay in week 20 (F_3,108_ = 385.99; *p* = 0.026, 0.016, and 0.011, respectively; Figure 6). For early-lay at week 25, CP and LP hens had the shortest TI durations compared to EP and NP hens (F_3,108_ = 246.36; *p* = 0.011), while at peak-lay in week 37, CP and LP hens had the shortest TI durations compared to EP hens (F_3,108_ = 222.58; *p* = 0.031), with NP hens having longer TI durations than EP hens (*p* = 0.037; Figure 6). By treatment per week, CP and EP hens showed shorter TI durations at week 37 compared to 20 and 25 (F_2,81_ = 126.89; *p* = 0.031), and LP hens showed shorter TI durations at week 25 and 37 compared to 20 (*p* = 0.033, and 0.027, respectively; Figure 6). No differences were found between weeks of testing for NP hens (*p* > 0.05; Figure 6). 

#### 3.2.2. Tonic Immobility Induction Attempts

**Figure 7:**
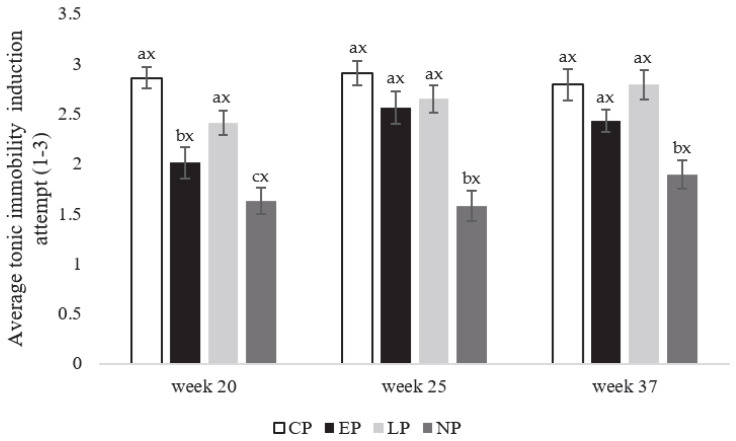
Tonic immobility induction attempts (1–3) expressed as (mean ± SEM) for laying hens in CP (continuous perch), EP (early perch), LP (late perch), and NP (no perch) housing environments during the onset of lay at week 21, early-lay at week 25, and peak-lay at 37 weeks of age (*n* = 112 hens/week). ^a–c^ Different superscripts indicate statistically significant differences between treatments within the same week at *p* < 0.05. ^x–z^ Different superscripts indicate statistically significant differences between weeks within the same treatment at *p* < 0.05.

At the onset of lay in week 20, attempts to induce TI were higher in CP and LP hens compared to EP and NP hens (F_3,108_ = 97.55; p = 0.029), with the lowest number of attempts to induce TI recorded in NP hens at week 20 (p = 0.036 (CP), 0.019 (EP), 0.027 (LP); Figure 7). At early-lay in week 25 (F_3,108_ = 88.59; *p* = 0.022) and peak-lay at week 37 (F_3,108_ = 102.95; p = 0.017), induction attempts were lowest in NP hens compared to other treatment groups; however, there were no observed differences in induction attempts between weeks within any treatment group (Figure 7).

### 3.3. General Summary of Results

Table 2 summarizes the current study’s AB and TI test results. 

## 4. Discussion

The objective of this study was to investigate the effects of early and late access to perches on anxiety and fearfulness in laying hens. Attention bias tests evaluate an animal’s level of anxiety, where shorter latencies to begin and resume feeding coupled with fewer vigilance behaviors indicate decreased anxiety compared to longer latencies to begin and resume feeding coupled with a greater occurrence of vigilance behaviors [33,34,35]. Tonic immobility tests can be used as a tool to measure fearfulness in poultry [25,36,37], where shorter TI durations and more induction attempts indicate decreased fearfulness compared to longer TI durations and fewer induction attempts. Birds housed with continuous access to perches showed responses consistent with decreased anxiousness and fearfulness compared to the other treatment groups. Birds without access to perches consistently exhibited responses suggesting increased anxiousness and fearfulness compared to birds with access to perches. We observed a negative impact of removing perches in the EP pens on fearfulness at the onset of lay in week 20 and the early-lay period in week 25 of age and on anxiety at the onset of lay in week 21 of age. Lastly, there was a negative impact of adding perches in the LP pens on anxiety at 21 weeks of age and fearfulness at 20 weeks of age. 

### 4.1. Attention Bias

CP hens showed the shortest latencies to begin and resume feeding regardless of age, showed the greatest percentages of birds that began and resumed feeding at weeks 21 (onset of lay) and 37 (peak-lay), and exhibited the lowest vigilance behavior scores at the onset of lay. This suggests that birds from CP pens showed less bias towards the perceived threat and more attention to the positive stimulus, indicating a lower anxiety level compared to birds from other treatment groups. The exceptions were that at peak-lay, CP and LP hens had similar latencies to resume feeding and that at week 21, CP and LP had a similar percentage of birds begin feeding. The NP hens showed increased anxiety based on longer latencies to begin feeding at peak-lay and resume feeding at weeks 21 and 37 compared to all other treatment groups, a lower percentage of birds to begin feeding at week 21, and the highest vigilance behaviors scores at weeks 21 and 37. These longer latencies and increased vigilance behaviors indicate greater attention allocated toward the perceived threat (conspecific alarm call), suggesting a higher anxiety level than the other treatment groups. However, some results do not fully align with this statement. At the onset of lay, EP and LP birds had similar latencies to begin feeding and similar percentages of birds to resume feeding, and EP birds had a similar vigilance behavior score as birds from NP pens. At peak-lay, EP and NP pens had similar percentages of birds to begin and resume feeding. 

Providing laying hens with perches throughout their life offers birds the opportunity to fulfill a strong motivation to perch. Laying hens are highly motivated to perch, which is reflected in their willingness to push open heavier doors in order to gain access to a perch than to gain access to a sham perch that could not be used for perching [38]. Our results align with previous findings that providing complex environmental conditions reduces anxiety in laying hens [7], broiler chickens [29], and starlings [39]. Broilers housed in complex pens with perches, dust baths, and temporary enrichments showed shorter latencies to begin and resume feeding in an AB test compared to broilers housed in monotonous environments, indicating reduced anxiousness in the former [29]. In contrast, laying hens housed in conventional cages showed responses indicating reduced anxiety compared to laying hens housed in floor pens with perches [7]. Although the methodologies were similar, latencies to begin and resume feeding were much lower in the previous study (54-100s for conventional cage and 54-146s for enriched floor pen in the previous study compared to 290–297 s for NP and 190–203 s for CP in the present study). This could be due to strain differences [40], test age differences (30 weeks of age compared to 21 and 37 weeks during the present study) [7], or inherent differences in husbandry. Ultimately, our results are the first to suggest that Hy-Line Brown hens housed with access to multi-tier perches throughout their lifetime are less anxious at weeks 21 and 37 of age than those housed without perches. 

During the AB test in week 21 (onset of lay), hens from the EP group showed similar latencies to begin feeding to hens from LP and NP pens, similar latencies to resume feeding to hens from LP pens, a comparable percentage of birds to resume feeding to those observed in LP and NP pens, and a similar vigilance score to hens from NP pens. This could indicate that the removal of perches increased anxiousness in hens from EP pens comparable to the addition of a novel object within the environment or having no perches at all. Previous research has established hens’ strong motivation to perch [18,38,41,42,43]. Chicks begin to perch between 7 and 10 days of age [43] and the amount of time spent perching increases with age [41]. By preventing access to perches during the lay phase, for which hens have an inelastic demand (they will work for access to perches despite increasing costs), hens may suffer and experience elevated levels of anxiety [44]. Depriving hens of the opportunity to perch after access to perches during rearing (0–17 weeks of age) can increase anxiety at the onset of lay (21 weeks of age). 

At peak-lay, birds from EP pens exhibited greater anxiety (longer latencies to resume feeding and fewer birds that began and resumed feeding) compared to birds from LP pens. However, some behavioral responses indicate decreased anxiety in the EP group compared to LP birds and NP birds (latency to feed), or similar levels of anxiety to LP birds (vigilance) and NP birds (percent of birds feeding). We would expect birds from EP pens to show increased anxiety at week 37 of age compared to birds from LP pens because they lack access to an appropriate environmental structure to exhibit perching behavior. Preventing the expression of this highly motivated behavior likely influences anxiety because hens do not have access to appropriate elevated surfaces which they perceive as a safe space, increasing the occurrence of negative states [45,46]. Furthermore, birds from EP pens showed decreased anxiety at peak-lay compared to birds from NP pens (latency to begin and resume feeding, vigilance behavior), suggesting that perch access, even when removed at 17 weeks of age, is more beneficial to anxiousness at 37 weeks of age than not having access to perches at all.

Late access to perches (LP) resulted in longer latencies to begin and resume feeding, greater vigilance, and fewer birds resuming feeding in week 21 compared to peak-lay. These responses indicate greater anxiousness at the onset of lay, when birds recently gained access to perches, compared to peak-lay when birds had prolonged perch access. Furthermore, providing late access to perches (LP) resulted in almost equally negative affective states compared to hens reared without perch access (NP) at week 21 of age (onset of lay), indicated by similar percentages and latencies of birds to begin feeding. The LP hens may still be adapting to their new environment, contributing to the responses consistent with increased anxiousness during week 21 (i.e., after the perches were added to the pens). Without any prior exposure to multi-tier perches during development, the hens may have experienced reduced spatial navigation skills, impairing their ability to successfully utilize the perches. For example, pullets reared with perches from 0-8 weeks of age were able to jump to higher perches compared to those without access to perches until after 8 weeks of age [13]. Additionally, there are concerns about transferring cage-reared pullets to aviaries due to their lack of navigational practice in a setting with greater vertical space [47]. Accidents during take-off to perch or landing are more common in birds reared without perches, which could increase the occurrence of keel bone fractures or collisions with pen mates resulting in aggressive interactions [47]. Furthermore, hens that did not receive enrichment in floor pens during rearing and were moved into an aviary at 25 weeks of age did not occupy the upper tiers of the aviary and took 20 weeks to adapt to the system [48]. Ultimately, pullets should be reared in conditions similar to their adult environment, likely also because this reduces behavior-related problems [47]. Although we did not measure perching behavior in the current study, hen responses during the attention bias test following the addition of perches within the environment indicated that hens took at least 16 weeks to adapt to their new environment, as they had no prior experience with perches. However, as we did not test between 21 and 37 weeks of age, future studies should focus on this period to discover the true adaptation period of hens to new objects within their home environment.

Adding perches later in life did not improve affect but rather had a varied result on behavioral responses during the AB test. At week 37 of age, birds from LP pens exhibited latencies to resume feeding that were comparable to hens from the CP group (LP: 66 s; CP: 48 s). However, LP hens began feeding later and had fewer birds begin and resume feeding than the CP treatment group at week 37. This could suggest that the addition of perches did not completely improve the affective state to the standard found in hens from the CP group, possibly because hens from LP pens did not have access to perches during musculoskeletal development, as did hens from CP pens. In other words, the quality of perch use was maybe insufficient as in the CP group because learning to use perches after the pullet phase takes longer due to low muscle strength, a lack of motor skills, and an inability to keep balance [13]. When looking at the within-treatment differences across weeks 21 (onset of lay) and 37 (peak-lay) for LP birds, there is a decrease in latencies to begin and resume feeding, as well as an increase in the percentage of birds to resume feeding, suggesting that the addition of perches did reduce anxiousness within the LP treatment group at peak-lay in week 37 of age. Another explanation may be based on the affective state as an accumulation of experiences. Affective states are the result of cumulative life experiences, ranging from positive to negative, and this can impact how animals respond to certain situations, specifically how anxiously an animal responds to perceived threats [34,49,50]. Hens from LP pens inherently had fewer positive experiences as they had fewer opportunities to express highly-motivated perching behavior than hens from CP pens that had perches their entire life. Subsequently, hens from LP hens were likely in a more negative affective state compared to hens from CP pens, inducing the bias towards potentially threatening stimuli during the AB test [34,50,51]. 

Overall, hens from CP pens showed decreased anxiety compared to other treatment groups at weeks 21 and 37 of age. Hens from NP pens consistently showed increased anxiousness at weeks 21 and 37 of age compared to hens from other treatment groups. Removing perches from the environment increased anxiety levels at 21 weeks of age; however, the effect of removing perches on anxiety levels at week 37 of age remains unclear. Adding perches to the environment (LP) resulted in slightly increased anxiety at 21 weeks of age; however, at week 37 of age, the anxiety level had decreased. 

### 4.2. Tonic Immobility

CP hens exhibited the shortest TI durations compared to other treatments across all weeks of testing, with the exception of early-lay at week 25 and peak-lay at 37 weeks of age, when durations did not differ from LP hens. Additionally, CP hens had the highest number of attempts to induce TI at the onset of lay in week 20 compared to EP and NP hens and at weeks 25 and 37 compared to NP hens. These results indicate that hens from the CP pens were the least fearful at week 20 of age and that hens from CP and LP hens were least fearful at weeks 25 and 37 of age, in alignment with some previous studies [7,52]. Laying hens in enriched pens with access to perches exhibited reduced TI durations compared to hens housed in conventional cages, suggesting they were less fearful [7]. Additionally, hens with access to perches from 16 to 74 weeks of age had a reduced flight distance compared to those without perches, indicating reduced fearfulness in the former and supporting the idea that access to perches improves the birds’ sense of security [52]. Other studies found no relationship between perch access and fearfulness [53,54]. For example, TI durations for laying hens housed with or without perches did not differ (232 s vs. 304 s) at 36 weeks of age [54]. These TI durations were longer than those observed in the current study at week 37 of age (CP: 31s vs. NP: 86 s), which could be attributed to genetic strain differences, different environments, or a different level of human interaction, as the TI methodology was comparable between studies. Domestic fowl selected for specific traits typically possess different temperaments, which can be shown through their level of fear or flightiness [54,55,56,57]. Our results suggest that providing Hy-Line Brown hens with multi-tier perches throughout their lifetime reduces fearfulness compared to all treatments at the onset of lay and compared to EP and NP treatments at early-lay and peak-lay.

Hens without perch access had longer TI durations and fewer induction attempts than hens with continuous or late perch access across all testing weeks, indicating they were more fearful. Perching is a natural behavior seen in domestic hens’ wild ancestors to avoid predation and remains a highly motivated behavior in laying hens even after years of domestication [38,58]. Allowing access to perches can reduce fearfulness, as birds gain a feeling of security from perching because they provide an unobstructed view of their surroundings [59,60]. Environments that do not provide appropriate perching structures may subject hens to increased fearfulness, as they may feel less secure due to their reduced surveillance of the surrounding area [58]. In line, laying hens on a low perch were quicker to escape due to an approaching ground predator than laying hens on an elevated perch, indicating that hens on higher perches have a better sense of security [46]. Our results suggest that hens without access to perches had a reduced sense of security and were more fearful than hens from CP and LP groups at weeks 20 and 25 and all treatment groups at peak-lay; however, no differences in fearfulness were found between EP and NP groups at the onset of lay in week 20 and early-lay at 25 weeks of age. 

After the removal of perches, TI durations for EP hens did not differ from NP hens at weeks 20 (EP: 91 s; NP: 84 s) or 25 (EP: 83 s; NP: 81 s) of age, suggesting that EP hens had similar levels of fear as hens without perches. This result could indicate the negative impact of the removal of perches at weeks 20 and 25 of age; however, by week 25, EP hens had a similar number of induction attempts as CP and EP hens. The removal of environmental structures important for performing highly motivated behaviors can have detrimental effects on animal welfare. For example, removing environmental enrichment resulted in a pessimistic judgment bias in starlings [61]. However, by week 37, EP hens had shorter TI durations compared to NP hens (EP: 65 s; NP: 86 s), indicating that EP hens adapted to the loss of resources by 37 weeks of age. This is further supported by the shorter TI durations with increasing age (week 37 vs. 20 and 25) in the EP treatment. While we did not observe differences in TI duration between EP and NP treatment groups during weeks 20 and 25, hens from the EP groups required a consistently higher number of attempts to induce tonic immobility compared to hens from NP groups, and thus it was more difficult to generate the anti-predator freezing response in hens from EP pens. This finding could indicate that providing perches only during rearing impacts fearfulness slightly less negatively than not providing perches at all.

Hens from LP pens were less fearful than hens from EP and NP pens at 20 (onset of lay), 25 (early-lay), and 37 (peak-lay) weeks of age but showed comparable fear to CP hens at weeks 25 and 37, suggesting that current perch access is more important than past access and better than no access to perches. Within the LP treatment group, TI durations were longer during week 20 compared to weeks 25 and 37. This indicates that adding perches early or late in life reduces fear, when fear is measured concurrent with perch access. However, this reduction in fear over time may also be due to repeated exposure to human presence. Although previous studies recommend rearing pullets in the same environment that they are destined for in the lay phase because of the influence that perch access during rearing has on adult behavior and spatial navigation skills while using perches [13,14], our results suggest that current access to perches reduces fear. We did not evaluate perching behavior in the current study, but whether the hens utilized the perches successfully or not, we still observed the beneficial effect of adding perches at 17 weeks of age on fearfulness at 20, 25, and 37 weeks of age.

Fear was greater in CP hens at the onset of lay and early-lay compared to peak-lay at week 37 of age. We argue that repeated exposure to humans that is inherent with husbandry conditions reduces fear as hens aged. While domesticated poultry are inherently afraid of humans [37,62], repeated exposure can reduce this fearfulness, especially when the interaction is considered to be positive [63,64,65]. Birds in the current study were exposed to human presence on a daily basis, and on many occasions, workers were inside the pens multiple times per day. It is possible that as the birds aged, they became increasingly habituated to human presence and handling, resulting in reduced fearfulness during the TI test. Although all hens were exposed to the same level of human interaction, CP, EP, and LP hens showed decreased fear responses as they aged, while NP hens did not. Hens from NP pens showed consistently longer TI durations compared to the other treatment groups. This could suggest that no access to perches: 1) was so impactful on the level of fear that habituation to human exposure made no difference, or 2) the inability to escape to a safe area hindered their ability to cope with human interaction. In line, laying hens seek out perches as a safe space from predators or aggressive pen mates, especially at night, for resting and to monitor their surroundings [18,58]. So, preventing access to perches negatively impacted fear in Hy-Line Brown laying hens. Overall, our results indicate that perch provision, either continuous or later in life, reduces fear when measured during perch access in Hy-Line Brown laying hens at early-lay and peak-lay.

Some previous work supports that anxiety and fear can be opposing and are different emotional experiences [3,7,27]. Where anxiety is a “coherent cognitive-affective structure” ultimately centered around the uncontrollability of possible future negative events, fear is an emotional response to presently dangerous negative events [66]. However, the behavioral responses to each can appear similar [67] and some studies have found anxiety and fear to be positively correlated as they are both coping strategies to escape from threats [40,68] This could be because there is overlap within the brain mechanisms controlling fear and anxiety, leading to the idea that anxiety is an exaggerated form of fear that allows the animal to prepare for future events [21]. In the current study, behavioral responses of anxiety and fearfulness did align with one another, although they likely produced different emotional experiences. 

Our study is limited by our solely behavioral measures of affective state. A truly well-rounded evaluation of affective state and animal welfare includes not only behavioral but also physiological (i.e., heart rate, blood pressure, heterophil lymphocyte ratio) measures. Future research should be conducted to confirm our findings that perch access can reduce both fear and anxiety in behavioral and physiological measures, including in different genetic strains.

## 5. Conclusions

The current study implies that providing laying hens with multi-tier perches throughout their lifetime can improve emotion and affective state by reducing fearfulness and anxiety, whereas no access to perches negatively impacted emotion and affective state. The addition of perches to the environment at 17 weeks of age resulted in greater anxiety at 21 weeks of age, but this effect decreased by 37 weeks of age, indicating that adaptation to a new adult environment requires at least 16 weeks. Furthermore, adding perches reduced fearfulness by week 20 of age compared to hens that lost their perch access or never had perch access. At weeks 25 and 37 of age, late access to perches resulted in similar fear levels as in hens with perch access their entire life, suggesting that current perch access reduces fearfulness. Removing perches from the environment at 17 weeks of age resulted in increased anxiety at 21 weeks of age and increased fearfulness at weeks 20 and 25 of age, which dissipated by week 37 of age. Furthermore, birds from EP pens showed decreased anxiety at week 37 compared to birds from NP pens, suggesting that perch access, even when removed at 17 weeks of age, is more beneficial to anxiousness at 37 weeks of age than not having access to perches at all. Our results indicate that continuous access to perches or access to perches at the time of assessment (for late access) resulted in the best outcomes for fear and anxiety in these laying hens. 

## Figures and Tables

**Table 1:** Summary of the attention bias (AB) testing methodology adapted from Campbell et al. [7]. Birds were tested in groups of three at 21 and 37 weeks of age.

Scenario	Procedure	Test Duration	Variables collected
Test begins	Play first alarm call	300 s	Not applicable
No birds begin feeding	Test runs for 300 s	300 s	All birds receive a maximum latency to begin feeding score of 300 s
One bird begins feeding	Test runs for 300 s	300 s	Latency to begin feeding for bird that began feeding Other two birds receive a maximum latency score of 300 s
Two birds begin feeding	Test runs for 300 s. Play second alarm call at 300 s and test runs for an extra 120 s.	420 s	Latencies to begin feeding for the two birds that began feeding Third bird receives a maximum latency score of 300 s Latencies to resume feeding for two birds that began feeding if they resume feeding before test ends
Three birds begin feeding before 270 s	Test runs until the last bird begins feeding. Allow birds 5 s to feed, then play second alarm call. Test runs until 300 s.	300 s	Latencies to begin and resume feeding for all three birds if they resume feeding before the test ends
Three birds begin feeding between 270–300 s	Test runs until the last bird begins feeding. Allow birds 5 s to feed, then play second alarm call. Test runs an extra 120 s.	420 s	Latencies to begin and resume feeding for all three birds if they resume feeding before the test ends

**Table 2:** Simple summary of attention bias (AB) and tonic immobility (TI) results. Hens were kept in continuous perch (CP), early perch (EP), late perch (LP), or no perch (NP) housing environments.

Attention Bias Test Measure					
Between Treatments	Latency to Begin Feeding (s)	Percent of Birds to Begin Feeding (%)	Latency to Resume Feeding (s)	Percent of Birds to Resume Feeding (%)	Vigilance Behaviors
Week 21	CP < EP, LP, NP	NP, EP < CP, LP	CP < EP, LP < NP	EP, LP, NP < CP	CP < LP < EP, NP
Week 37	CP < EP < LP < NP	NP, EP < LP < CP	CP, LP < EP < NP	NP, EP < LP < CP	CP, EP, LP < NP
Between weeks					
CP	Not sig. between weeks	Not sig. between weeks	Not sig. between weeks	Not sig. between weeks	Not sig. between weeks
EP	Week 37 < week 21	Week 37 < week 21	Week 21 < week 37	Not sig. between weeks	Week 37 < week 21
LP	Week 37 < week 21	Not sig. between weeks	Week 37 < week 21	Week 21 < week 37	Not sig. between weeks
NP	Not sig. between weeks	Not sig. between weeks	Not sig. between weeks	Not sig. between weeks	Not sig. between weeks
Tonic Immobility Measure					
Between treatments	Duration (s)			Induction attempts	
Week 20	CP < EP, LP, NP			NP < EP < CP, LP	
Week 25	CP, LP < EP, NP			NP < LP, EP, CP	
Week 37	CP, LP < EP < NP			NP < LP, EP, CP	
Between weeks					
CP	Week 37 < week 20, 25			Not sig. between weeks	
EP	Week 37 < week 20, 25			Not sig. between weeks	
LP	Week 25, 37 < week 20			Not sig. between weeks	
NP	Not sig. between weeks			Not sig. between weeks	

## Data Availability

For access to data from the study, please contact the corresponding author.
